# Comprehensive evaluation of environment adaptability in wild and captive lenok (*Brachymystax lenok*): from the perspective of antioxidant capacity, immune response and gut microbiome

**DOI:** 10.3389/fmicb.2026.1764670

**Published:** 2026-03-04

**Authors:** Luye Bai, Ziyang Wang, Hongxing Wang, Bo Ma

**Affiliations:** 1 College of Fisheries and Life Science, Dalian Ocean University, Dalian, China; 2 Heilongjiang River Fishery Research Institute, Chinese Academy of Fishery Sciences, Harbin, China; 3 College of Life Science and Technology, Harbin Normal University, Harbin, China; 4 College of Fisheries and Life Science, Shanghai Ocean University, Shanghai, China; 5 Research Station for Field Scientific Observation on Fishery Resources and Ecological Environment Protection, Jiamusi, Ministry of Agriculture and Rural Affairs, Jiamusi, China

**Keywords:** biodiversity restoration, *Brachymystax lenok*, digestion, gut microbiota, immunity

## Abstract

**Introduction:**

The intestinal microbiota is considered an adaptive trait closely associated with reintroduction success and may contribute to the ecological fitness of *B. lenok*.

**Methods:**

In this study, intestinal morphology, digestive enzyme activity, immune parameters, and gut microbiota composition were compared between wild and farmed *B. lenok* to elucidate differences in intestinal and hepatic health under distinct aquatic environments.

**Results:**

Histological analysis showed that villi in the hindgut of wild individuals were longer and denser than those of farmed ones. Although the intestinal structure of farmed *B. lenok* remained intact, their villus morphology and density differed significantly from those of the wild group. Compared with the farmed group, wild B. lenok showed higher hepatic immune/antioxidant activity (elevated alkaline phosphatase (AKP), acid phosphatase (ACP), lysozyme (LYZ), and catalase (CAT), as well as glutathione (GSH) content) and up-regulated liver immune-related genes (*c3*, *foxo1*, *igM*, *il-10*, *lyz*, etc.), while farmed fish displayed higher intestinal stress markers (CAT, malondialdehyde (MDA) and a pro-inflammatory signature (*il-6*, *il-1*β upregulated). Microbiota profiling revealed higher abundance of Firmicutes and Bacteroidetes but a trend of decreasing Proteobacteria in the wild group.

**Discussion:**

Collectively, these findings demonstrate significant differences in intestinal morphology, digestive function, and microbial community composition between wild and farmed *B. lenok*. This study provides new insights for improving post-stocking adaptability in reintroduction programs and proposes novel conservation strategies for biodiversity restoration.

## 1 Introduction

*Brachymystax lenok* belongs to the order Salmoniformes, family *Salmonidae*, and genus *Brachymystax*. It is widely distributed in the Siberian region of northern Asia, from the Ertis–Ob river basin eastward to the Kolyma River and Primorsky Krai in Russia (Froufe et al., 2008; Osinov, 2024). In China, *B. lenok* primarily inhabits the Heilongjiang River in the northeast and the Ertis River basin in the northwest. As a cold-water species, *B. lenok* typically resides in clean, fast-flowing mountain streams with low water temperatures, showing high sensitivity to environmental changes. Owing to its genetic diversity and ecological specificity, it serves as a valuable model species for studies on fish population genetics and phylogeographic structure. In recent years, the wild germplasm resources of *B. lenok* have declined sharply due to climate warming, environmental pollution, and overfishing, and the species is now on the brink of extinction. It has been listed as a National Class II Key Protected Wild Animal in China since 2021. Consequently, artificial propagation under controlled conditions has become a crucial measure for conserving the remaining populations. However, multiple attempts to reintroduce hatchery-bred individuals into natural habitats have achieved limited success (Bouchard et al., 2022; McMillan et al., 2023), with one potential explanation for this being the loss of wild-type adaptive traits in captive-bred fish (Willoughby et al., 2015). Loss of wild-type adaptive traits refers to the collective changes in genetic, physiological, behavioral, and microbial characteristics that together determine an organism’s fitness in its natural environment. Among these, gut-microbiota composition is increasingly recognized as an independent, heritable adaptive trait; recent studies show that probiotic or microbiome conditioning significantly enhances the survival rate and ecological fitness of reintroduced fish (Ma et al., 2025; Kanika et al., 2025).

The intestine in fish is not only a major organ for digestion and nutrient absorption, but also the largest interface between the organism and its external environment. The gut microbiota performs various beneficial functions for the host, including nutrient acquisition, immune regulation, and developmental modulation, enabling adaptation to lifestyle changes (Gao et al., 2023; Wang Q. et al., 2025). The composition and diversity of the intestinal microbiota are influenced by numerous factors, such as diet, stress, and health status (Singh et al., 2025; Sun et al., 2024). For example, captive animals often have diets that are higher in carbohydrates and lower in fiber compared to their wild counterparts, which can lead to significant differences in gut microbial composition. Additionally, the higher stress levels in captive environments can also alter the gut microbiota, potentially affecting overall health. Previous studies have revealed distinct microbiota profiles between wild and hatchery-reared fish, suggesting that the physiological state of a host may be regulated by its gut microbial community (Kanika et al., 2025). We hypothesize that these microbiota differences could reduce post-release performance of hatchery fish. Therefore, comparative studies of the gut microbiota between wild and captive animals can help identify key microbial features associated with successful adaptation to natural environments.

The digestive performance of fish is closely related to the absorptive capacity of the digestive organs (Ribeiro et al., 1999), which depends on the activity of digestive enzymes such as proteases, lipases, and amylases (Tengjaroenkul et al., 2000). Trypsin is the most important alkaline protease during early digestive development in fish, activating other pancreatic enzymes in the intestinal lumen. Amylase provides glucose energy, while lipase hydrolyzes dietary triglycerides into free fatty acids (Castro-Ruiz et al., 2021). Because digestive enzyme activities are rapidly up- or down-regulated by shifts in temperature, diet and flow regime, they directly constrain food conversion, energy storage and early growth (Navarro-Guillén et al., 2022; Grčić et al., 2023), all of which determine whether *B. lenok* survives after release. Comparing the change of these enzymes between hatchery and wild fish therefore provides a maker for post-release environmental adaptability.

In this study, we systematically compared the intestinal morphology, digestive enzyme activity, immune-related enzyme activity, immune gene expression, and intestinal microbial composition between wild and farmed *B. lenok* populations. There, based on differences in dietary fat content and our preliminary enzyme survey showing higher hepatic GSH and lower intestinal MDA in wild fish, we formulated the following a priori hypotheses: Firstly, wild fish will allocate more resources to hepatic immune and antioxidant systems, reflected by higher hepatic LYZ, CAT, and GSH levels. Secondly, farmed fish will exhibit elevated intestinal stress markers (MDA, CAT) due to high-fat feeding. Finally, the gut microbiome will shift toward carbohydrate-associated taxa under commercial feed, while wild fish maintain protein/lipid-adapted communities. Using then histological observations and high-throughput sequencing, we aimed to identify physiological and microbiological differences in intestinal and hepatic health. The findings of this study provide scientific evidence for optimizing rearing strategies and environmental regulation during artificial propagation, thereby advancing large-scale breeding and conservation of this endangered species.

## 2 Materials and methods

### 2.1 Fish sampling

Farmed *Brachymystax lenok* (F group: *n* = 15; body length: 36.90 ± 2.20 cm; body weight: 771.88 ± 29.78 g; Age (years): 5^+^) were obtained from the Bohai Cold-Water Fish Experimental Station, Heilongjiang Fisheries Research Institute, China. Wild *B. lenok* (W group: *n* = 15; body length: 35.10 ± 6.63 cm; body weight: 777.23 ± 21.51 g; Age (years): 4^+^) were captured in October 2024 from the upper reaches of the Huma River, Heilongjiang Province. All farmed fish were derived from the same broodstock population and fed commercial pelleted diets; the nutrient composition of the feed is presented in Supplementary Table S1. Water quality parameters of wild and farmed environments were as shown in Supplementary Table S2. Ten individuals from each group were randomly selected, anesthetized with MS-222 (Sigma, United States), and immediately placed on ice for dissection. All procedures were conducted in accordance with the Directive 2010/63/EU on the protection of animals used for scientific purposes. Under aseptic conditions, the liver, pyloric caeca, stomach, foregut, midgut, and hindgut were dissected. Part of each tissue was stored in a sterile Eppendorf tube, flash-frozen in liquid nitrogen for 2 h, and then kept at -80°C for subsequent analyses, including diet examination, biochemical assays, qRT-PCR, and intestinal microbiota profiling. Another portion of the tissue was fixed in 4% paraformaldehyde for histological observation.

### 2.2 Observation of feeding habits

To investigate the feeding habits of *B. lenok*, the stomachs of the experimental fish were removed and opened immediately after dissection. The stomach contents were transferred into Petri dishes containing 5 mL of physiological saline as a diluent. Visible prey items were classified by type, and the weight of each food category were recorded. Unidentifiable components, degraded by gastric digestion, were examined using a digital microscope (S6D, Leica, Germany) at 40 × magnification to determine prey types. For gastric contents degraded beyond visual identification, total DNA was extracted using the Ezup column kit (Sangon Biotech, Shanghai, China), amplified by PCR using universal primers (LCO1490: 5′-GGTCAACAAATCATAAAGATATTGG-3′; HCO2198: 5′-TAAACTTCAGGGTGACCAAAAAATCA-3′) (Xu et al., 2023). The PCR amplification was performed using the following thermal cycling parameters: 95°C for 180 s, followed by 40 cycles of 95°C for 5 s, 60°C for 15 s (annealing temperature), and 72°C for 30 s. The amplified products were then Sanger-sequenced (Sangon Biotech, Shanghai, China). The sequencing data were compared with reference sequences in the GenBank database (Release 262.0, August 15, 2024) for precise taxonomic identification of stomach contents.

### 2.3 Histological observation of intestinal tissues

To assess differences in intestinal structure between wild and farmed *B. lenok*, standard hematoxylin–eosin (H&E) staining was performed. For each fish, three 0.25 cm segments were taken from the foregut, midgut, hindgut, respectively (nine segments per individual in total). Briefly, liver and intestinal samples were fixed in 4% paraformaldehyde for 24 h, replaced with fresh fixative, and post-fixed for an additional 96 h. The samples were dehydrated through a graded ethanol series (75–100%), cleared in xylene, and embedded in paraffin. Sections of 5 μm thickness were prepared using a microtome (HM 325, MICROM, Germany), mounted on slides, and stained following the manufacturer’s protocol. After drying, sections were observed under a light microscope (Eclipse Ci-L, Nikon, Japan), and images were captured with a digital camera. From each section, five intact, well-oriented villi were randomly selected and measured. Morphometric measurements including muscularis thickness, villus height and width, submucosa thickness, and lamina propria width were analyzed using Image-Pro Plus 6.0 software.

### 2.4 Enzyme activity assays

Commercial assay kits from Shanghai Jianglai Biotechnology Co., Ltd. (Shanghai, China) were used to quantify enzyme activity following the manufacturer’s instructions. The following enzymes and parameters were measured: Trypsin (JL-T1273), amylase (JL-T0701), lipase (LPS, JL-T1341), acid phosphatase (ACP, JL-T1094), alkaline phosphatase (AKP, JL-T0946), lysozyme (LYZ, JL-T1062), superoxide dismutase (SOD, JL-T0781), catalase (CAT, JL-T0900), glutathione (GSH, JL-T0906), and malondialdehyde (MDA, JL-T0761). Liver, stomach, pyloric caeca, and intestinal tissues were homogenized in chilled extraction buffer at a 1:9 (w/v) ratio. The homogenates were centrifuged at 12,000 rpm for 10 min at 4°C, and supernatants were collected for analysis. Optical density (OD) was determined using a microplate reader (SpectraMax Plus 384, Molecular Devices, United States) at the following wavelengths: 540 nm (amylase), 405 nm (trypsin, LPS, AKP, ACP), 530 nm (LYZ), 560 nm (SOD), 510 nm (CAT), 412 nm (GSH), and 532/600 nm (MDA). Enzyme activity was calculated based on OD values and tissue wet weight.

### 2.5 RNA extraction, cDNA synthesis, and quantitative real-time PCR

Total RNA was extracted from liver and intestinal samples using TRIzol reagent (Thermo Scientific, United States) according to the manufacturer’s instructions. The quality (including purity and concentration) of RNA was evaluated using a NanoDrop ND-1000 spectrophotometer (Thermo Scientific, Wilmington, United States), while the degradation and contamination levels of RNA were determined by 1% agarose gel electrophoresis. First-strand cDNA was synthesized using a PrimeScript™ RT reagent kit with a gDNA Eraser (TaKaRa, Japan). The qRT-PCR assays were performed to quantify the mRNA expression levels of *il-1β, il-6, il-10, tnf-α, tlr1, tlr3, myd88, nf-κb, foxo1, igM, lyz, c3, mpo*, and *ssa1*. Each reaction (10 μL total volume) contained 0.4 μL primers, 5 μL 2 × TB Green Premix Ex Taq II (with Tli RNase H Plus), 3 μL sterile distilled H_2_O (dH_2_O), 0.2 μL 50 × ROX Reference Dye II, and 1 μL cDNA template. The thermocycling parameters were as follows: initial denaturation at 95°C for 180 s, followed by 40 cycles of 95°C for 5 s, 60°C for 15 s, and 72°C for 30 s. Amplifications were conducted using an ABI 7,500 Real-Time PCR System (Thermo Fisher Scientific, United States). β-*actin* (F: 5′-GGACTTTGAGCAGGAGATGG-3′; R: 5′-ATGATGGAGTTGTAGGTGGTCT-3′) was used as an internal reference to detect the relative expression of the target genes. The primer sequences are listed in Table 1. Primer amplification efficiency and linearity were rigorously assessed via the slope (m) and coefficient of determination (*R*^2^) of the standard curve; only primer sets exhibiting a slope between -3.6 and -3.1 (corresponding to 90–110% efficiency) and an *R*^2^ > 0.98 were advanced to subsequent analyses. Specificity was verified by a single, distinct melt-curve peak and the exclusive presence of a single, target-sized amplicon on agarose gel electrophoresis. Relative gene expression levels were calculated using the 2^–ΔΔCT^ method, and all samples were analyzed in triplicate.

**TABLE 1:** Primer sequences used for qRT-PCR.

Gene	Primer sequence	Product length (bp)	Accession number
*c3*	F: TGTCTGAGGGTGTGCTGATTC R: TGTCTGGAACCCGATCAACTG	117	XM_042764266.1
*foxo1*	F: TGTGGCCTGATTCCCTTGAC R: TGGGGACTGTGGTTGTGATG	146	XM_021564372.2
*igM*	F: GCAAATACCCACAGTTCCGC R: GACAAACACCGAAGCACCTG	93	NM_000074.3
*il-6*	F: GCGCTCGTGGTGTTAGTTAAG R: ATCACTTTCTCCCACTTCGGG	134	NM_001124657.1
*il-10*	F: GCTCTCTCCTCCTGTCCCT R: ATGGTGGAGAAGGCGGTG	148	NM_001245099.1
*il-i*β	F: CCCTGGAGTCTGCCCATTAC R: GAATGTGGTGTTGCGGTTGA	113	XM_021622166.2
*lyz*	F: TCCTCGTGTGAAAGCAAGACA R: GAATCCCTCAAATCCATCAAGCC	84	NM_139180.1
*mpo*	F: CCCTCATCCAACCCTTCATGT R: TTACCTTCCAGCACGACCC	118	NM_000250.2
*myd88*	F: GGATTGCCAGGACCCAACA R: CACAACGTCCTTTCTGTCCAC	115	XM_036943663.1
*nf-kb*	F: GGTGGAAGAGATTTGGGGCA R: ATCATACATGGAAGGGTGGGAG	151	KU238083
*ssa1*	F:CGATGCCAGAGAGAATATCCAGA R: GTCGGAAGTGATTGGGGTCTT	113	NM_199161.5
*tlr1*	F: CTTGGTAGCCAGTTACGTGGT R: TCAGGTGCTTCCAGGTGATG	101	NM_001166101.1
*tlr3*	F: GGAACATAGGTGGAGAATGGGT R: ACTGAGAGGTGAGCTTGCTG	85	NM_001013269.3
*tnf*-α	F: ACGGTGATGCTGAGTCCAAA R: TCAGTCCACAGTTTGTCCCC	97	NM_001124357.1

### 2.6 16 S rRNA gene sequencing and gut microbiota analysis

Three and five intestinal content samples were selected from the farmed (F, *n* = 3) and wild (W, *n* = 5) groups, respectively, and DNA was extracted using an E.Z.N.A. Soil DNA Kit (Omega Bio-tek, United States). The purity and integrity of nucleic acids were verified with a NanoDrop 2000 spectrophotometer (Thermo Fisher Scientific Inc., United States). Subsequently, 16S rRNA gene (V3–V4) was amplified using primers 338F (5′-ACTCCTACGGGAGGCAGCAG-3′) and 806R (5′-GGACTACHVGGGTWTCTAAT-3′) on a PCR instrument (GeneAmp 9700, ABI, United States). The PCR reaction mixture including 4 μL 5 × Fast Pfu buffer, 2 μL 2.5 mM dNTPs, 0.8 μL each primer (5 μM), 0.4 μL Fast Pfu polymerase, 10 ng of template DNA, and ddH_2_O to a final volume of 20 μL. PCR amplification cycling conditions were as follows: initial denaturation at 95°C for 3 min, followed by 27 cycles of denaturing at 95°C for 30 s, annealing at 55°C for 30 s and extension at 72°C for 45 s, and single extension at 72°C for 10 min, and end at 4°C. Purified amplicons were pooled in equimolar amounts and paired-end sequenced on an Illumina NextSeq 2000 platform (Illumina, San Diego, United States) according to standard protocols by Majorbio Bio-Pharm Technology Co., Ltd. (Shanghai, China). Raw FASTQ files were demultiplexed using an in-house Perl script, quality-filtered with fastp v0.19.6, and merged with FLASH v1.2.7 under the following criteria: (i) reads truncated at Q < 20 over 50 bp; truncated reads < 50 bp or containing ambiguous bases were discarded; (ii) only overlaps ≥ 10 bp with ≤ 0.2 mismatch were merged; unmerged reads were discarded; (iii) exact barcode matching, ≤ 2 nt primer mismatch. Operational taxonomic units (OTUs) were clustered at 97% identity using UPARSE implemented in VSEARCH v2.7.1. Chimeras were removed with UCHIME3 (*de-novo*) and OTUs < 2 reads or > 1% primer mismatch were discarded. The representative OTU sequences were classified into organisms using a native Bayesian model, and the species composition was analyzed. A Venn diagram was used to identify unique and shared OTUs between the W and F groups at the phylum, family, and genus levels. Venn diagrams were generated to identify shared and unique OTUs between groups. Alpha diversity indices (Chao1, Shannon, Simpson) were calculated using QIIME software v1.8.0, while Beta diversity was assessed using the Bray-Curtis algorithm and visualized through Principal Coordinate Analysis (PCoA) and Principal component analysis (PCA). Functional prediction and Kyoto Encyclopedia of Genes and Genomes (KEGG) pathway analysis were conducted using PICRUSt2, and biomarker features were identified via LEfSe analysis.

### 2.7 Statistical analysis

Biochemical data were analyzed using Microsoft Excel and SPSS 22.0 software. All results are expressed as the mean ± standard deviation (SD). Prior to analysis, data normality and homogeneity of variance were verified using the Kolmogorov–Smirnov and Levene’s tests, respectively. Significant differences between groups were determined by independent-samples *t*-test. Differences were considered statistically significant at *P* < 0.05. DESeq2 (v1.34) was used on relative-abundance OTU tables; *p*-values were adjusted by Benjamini–Hochberg False discovery rate (FDR) (*q* < 0.05, | log*2*FC| > 1). Only FDR-significant taxa are reported as differential.

## 3 Results

Due to field-access and husbandry restrictions, the farmed group remained at *n* = 3, resulting in an unbalanced design. While rarefaction suggest the main conclusions are robust, the small sample size may limit external validity. Multi-cohort, seasonal replicates are needed in future work to confirm the generality of our findings.

### 3.1 Feeding habit authentication

Based on morphological and molecular identification, the primary prey items of wild *B. lenok* consisted primarily of benthic invertebrates (82.43%), among which mayflies, stoneflies, and caddisflies accounted for 68.40% (Supplementary Table S3). In addition, small quantities of terrestrial insects and fish were observed. These results indicate that *B. lenok* is a carnivorous species primarily feeding on benthic macroinvertebrates.

### 3.2 Intestinal histological characteristics of wild and farmed populations

**FIGURE 1:**
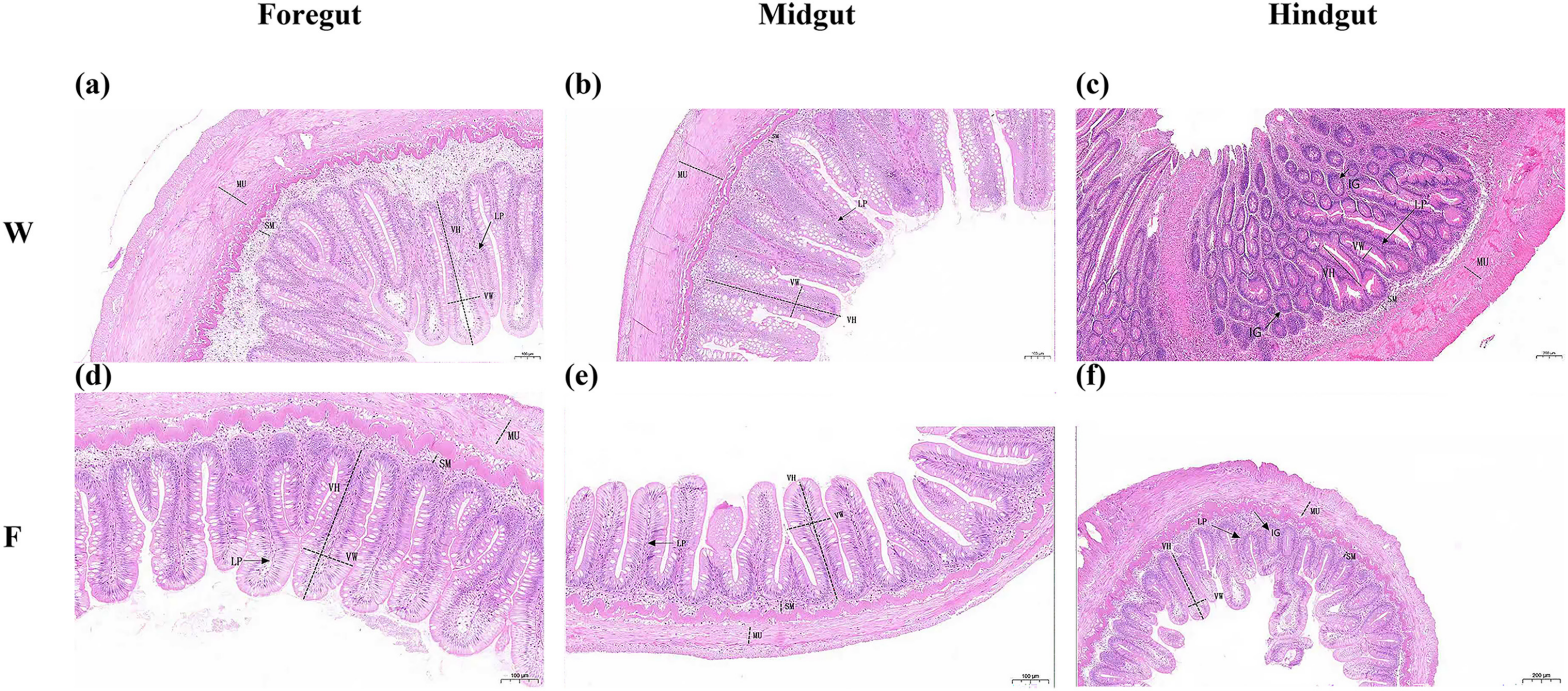
Histological observation of intestinal tissue structure in farmed and wild lenok (*Brachymystax lenok*). **(a–c)** Represent different gut tissues (foregut, midgut, and hindgut) from the captive group (Scale bar, 100 μm). **(d–f)** Represent different gut tissues (foregut, midgut, and hindgut) from the wild group. Scale bar, 100 μm **(d,e)**, 200 μm **(f)**. MU, muscularis; SM, submucosa; LP, lamina propria; IG, intestinal gland; VW, villi width; VH, villi height.

**FIGURE 2:**
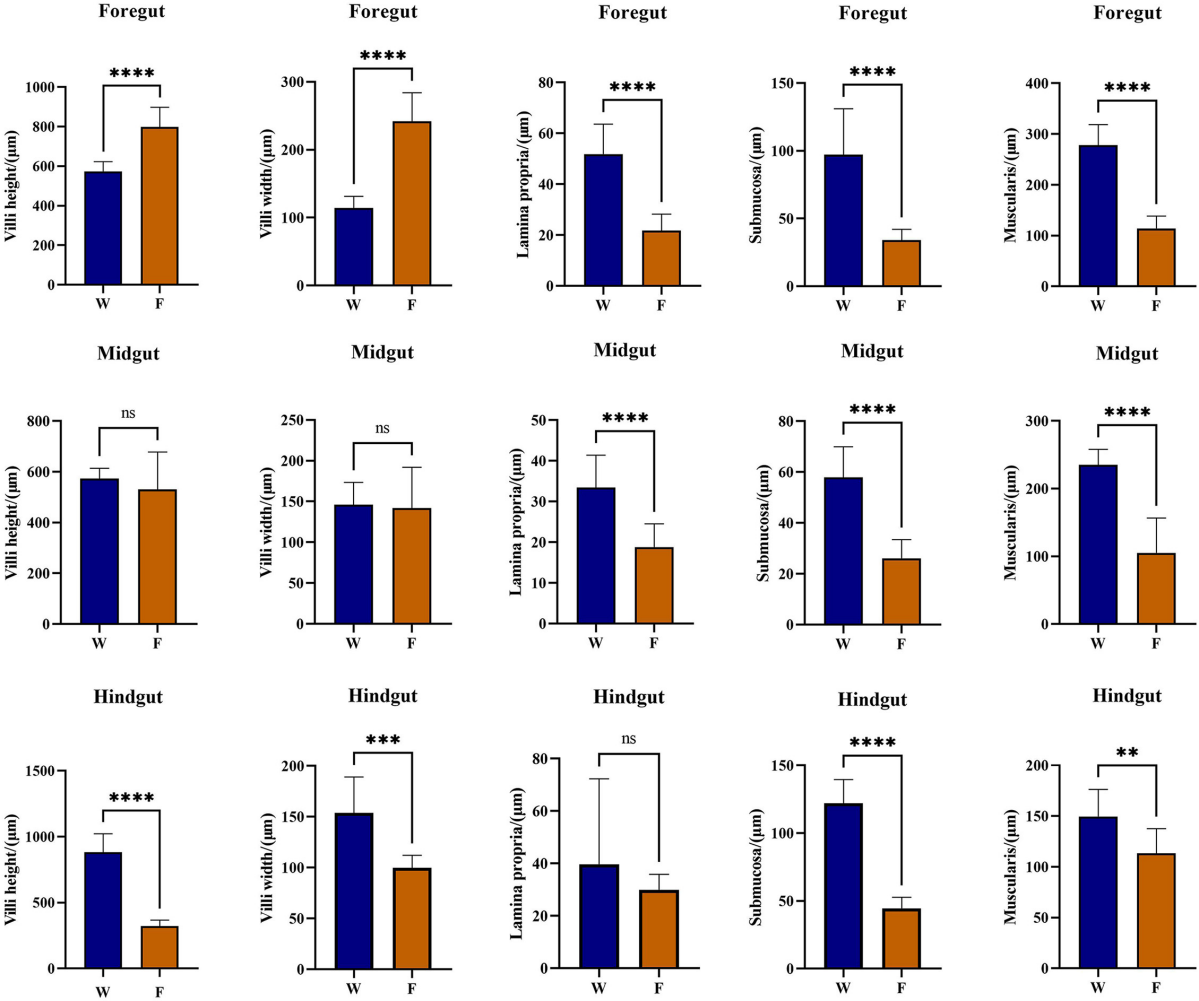
Morphological measurements of villi height, villi width, muscular thickness, thickness of submucosa, and lamina propria width, respectively (mean ± SD, *n* = 7). Independent-samples *t*-test with least significance difference *post hoc* test was used to compare the difference between two groups. Values marked with asterisks are significantly different (****P < 0.0001; ***P < 0.001; **P < 0.01).

Histological observations revealed that both wild (W) and farmed (F) *B. lenok* exhibited typical intestinal structures composed of four distinct layers: mucosa, submucosa, muscularis, and serosa (Figure 1). In farmed individuals, the foregut displayed intact structures with long and densely packed. The midgut featured a thinner muscularis layer but denser and more branched villi. The hindgut possessed the thinner muscularis layer, with short and narrow villi and more branches. In the wild group, the striated borders of the foregut, midgut, and hindgut were smooth and flat, the mucosal epithelial cells were arranged in an orderly manner, and the goblet cells were distributed uniformly. Based on microscopic observations, the villus length, villus width, muscle layer thickness, lamina propria width, and submucosa thickness of intestinal tissues in wild and farmed *B. lenok* were statistically analyzed (Figure 2). Compared with those in the farmed group, the villus length and width of the foregut in the wild group were significantly decreased (*P* < 0.0001), while those of the hindgut were significantly increased, and there was no significant difference in the midgut (*P* > 0.05). The lamina propria width, submucosa thickness, and muscle layer thickness of the foregut and midgut in the wild group were significantly higher than those in the farmed group (*P* < 0.0001). Similarly, the submucosa thickness and muscle layer thickness of the hindgut in the wild group were significantly increased compared with the farmed group (*P* < 0.01). Wild *B. lenok* consistently exhibited a thicker lamina propria, submucosa, and muscularis throughout the entire intestine, reflecting a structurally reinforced gut wall relative to farmed fish.

### 3.3 Comparison of digestive enzyme activity

**FIGURE 3:**
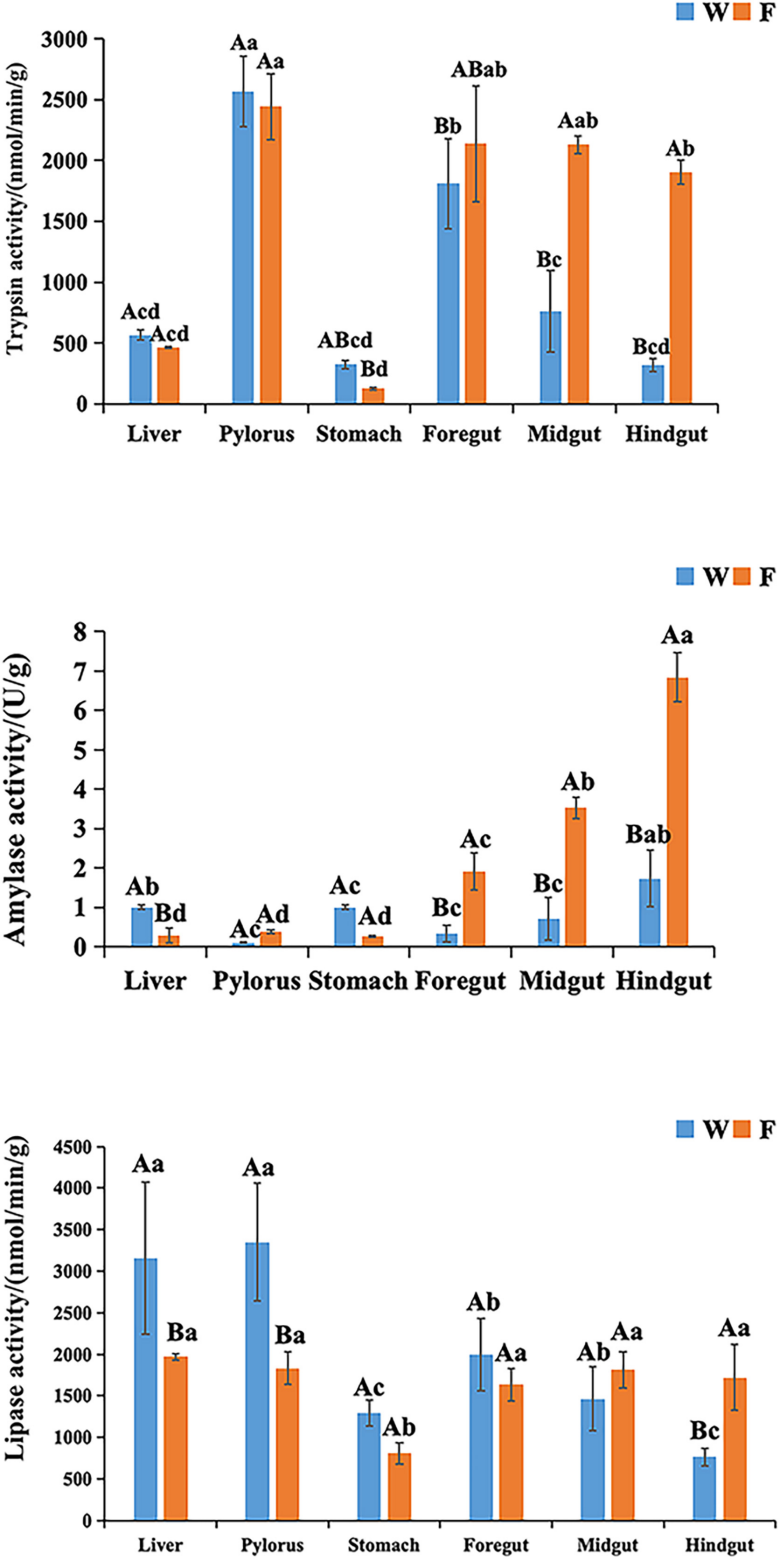
Digestive-related parameters of wild (W) and cultured (F) lenok (*Brachymystax lenok*). Results are shown as Mean ± SD (*n* = 5). Different small letters above the bars indicate significant differences (*P* < 0.05) at different tissues of same group and different capital letters above the bars indicate significant differences (*P* < 0.05) in different groups at the same tissues.

As shown in Figure 3, digestive enzyme activity patterns varied between wild and farmed *B. lenok*. Trypsin activity in the intestines of wild individuals was significantly lower than in farmed ones (*P* < 0.05), while gastric trypsin activity remained comparable between groups (*P* > 0.05). In both groups, trypsin specific activity was significantly higher in pyloric caeca and intestine than in all other tissues (*P* < 0.05). Amylase activity in the livers and stomachs of wild *B. lenok* was significantly higher than in the farmed group, but lower in intestinal tissues (*P* > 0.05). In both wild and farmed individuals, amylase activity reached its highest level in the hindgut. Lipase activity in the liver and pyloric caeca of wild *B. lenok* was significantly greater than in the farmed group, while activity in the hindgut was significantly lower (*P* > 0.05). In both populations, the lowest lipase activity occurred in the hindgut and stomach, respectively.

### 3.4 Analysis of immune enzyme activity

**FIGURE 4:**
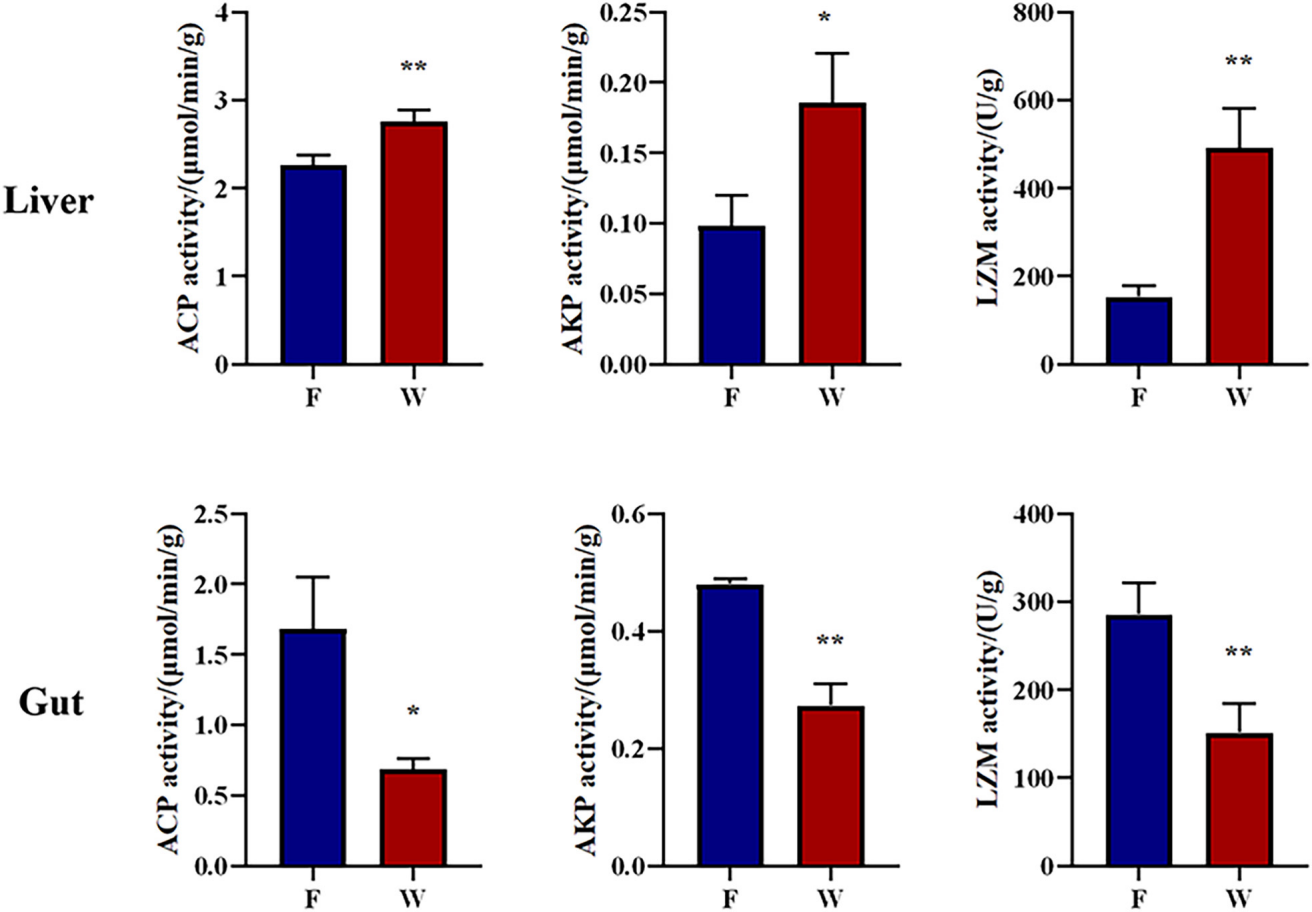
Intestinal and haptic immune related enzymes of the wild (W) and farmed (F) lenok (*Brachymystax lenok*). Results are shown as Mean ± SD (*n* = 5). Values marked with asterisks are significantly different (*P < 0.05; **P < 0.01).

Changes in the levels of ACP, AKP, and LYZ in liver and intestinal tissues are summarized in Figure 4. There were statistical differences in the activity of ACP, AKP, and LYZ in the liver and intestine between the wild and farmed groups (*P* < 0.05). The activity of ACP, AKP, and LYZ in the livers of the wild group were significantly higher than those in the livers of the farmed group (*P* < 0.05). On the contrary, the activities of ACP, AKP, and LYZ in the intestinal tissues of farmed *B. lenok* were significantly higher than those in the intestinal tissues of the wild group (*P* < 0.05). The Tissue × Origin interaction term was highly significant for all three enzymes (*P* < 0.001), indicating that the immune expression of *B. lenok* under different growth environments is tissue-specific.

### 3.5 Analysis of antioxidant parameters

**FIGURE 5:**
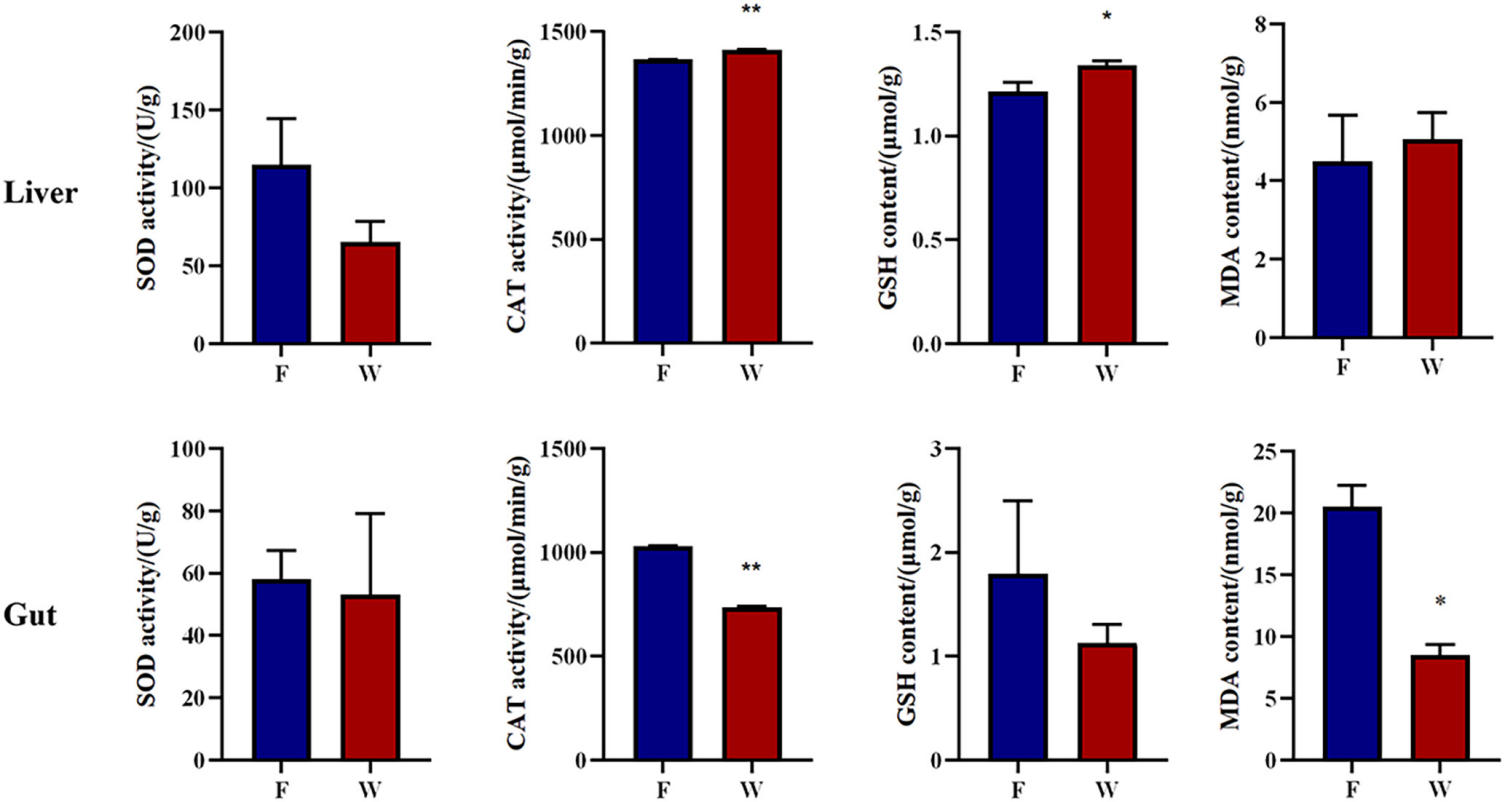
Intestinal and haptic antioxidant related parameters of the wild (W) and farmed (F) lenok (*Brachymystax lenok*). Results are shown as Mean ± SD (*n* = 5). Values marked with asterisks are significantly different (*P < 0.05; **P < 0.01).

As shown in Figure 5, hepatic CAT activity and GSH content in the wild group were significantly higher than in the farmed group (*P* < 0.01). However, in intestinal tissues, CAT activity and MDA levels were significantly higher in farmed *B. lenok* compared with wild individuals (*P* < 0.01). Additionally, stable water temperature, dissolved oxygen (DO), pH value and low ammonia levels ruled out water quality parameters as a driver of intestinal oxidation.

### 3.6 Expression of immune and inflammatory genes in liver and intestine

**FIGURE 6:**
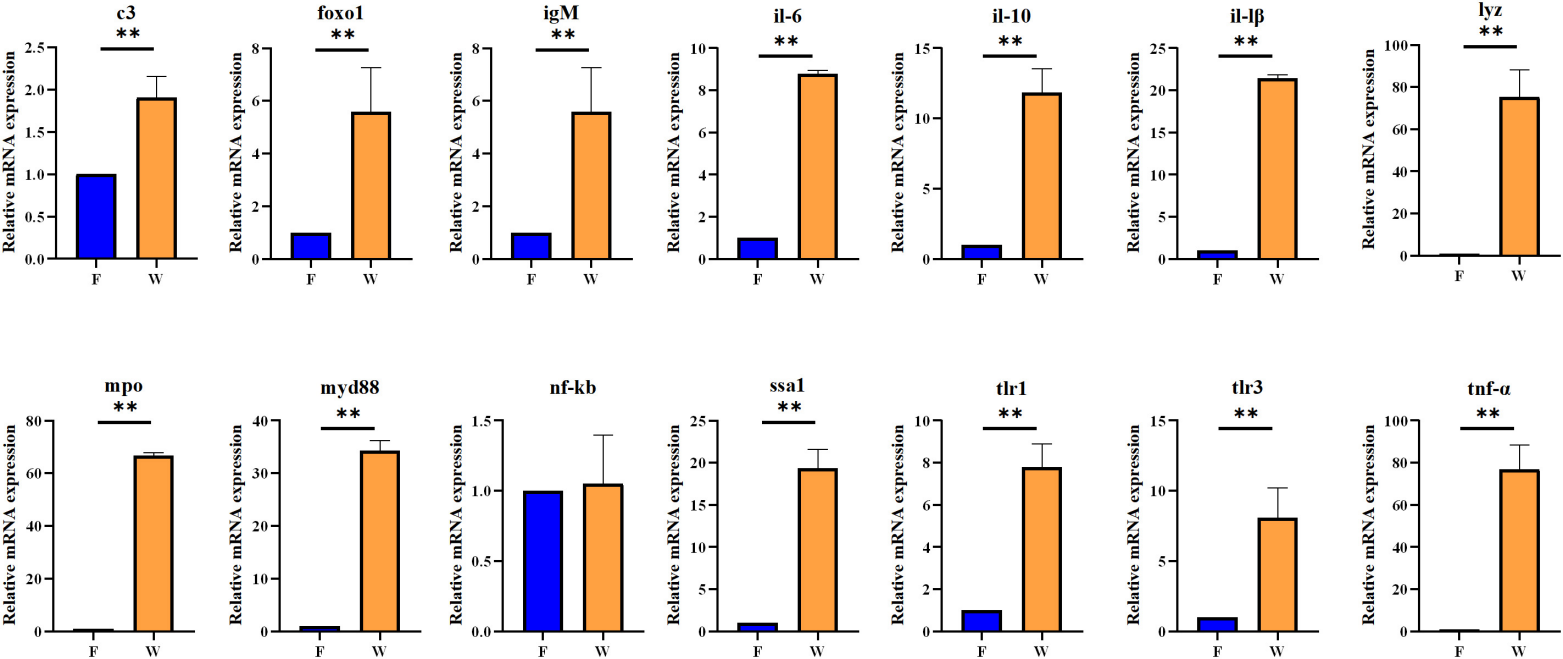
The mRNA expressions of immune- and inflammatory-related genes in liver tissue of the wild (W) and farmed (F) lenok (*Brachymystax lenok*). Results are shown as Mean ± SD (*n* = 5). Values marked with asterisks are significantly different (**P < 0.01).

**FIGURE 7:**
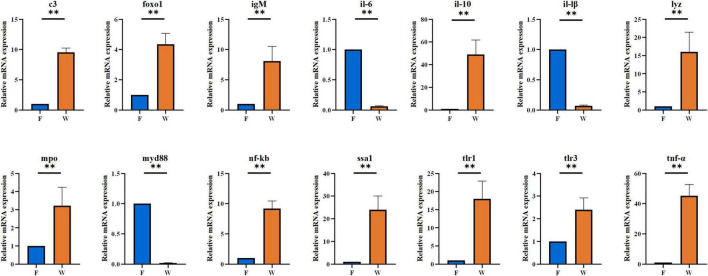
The mRNA expressions of immune- and inflammatory-related genes in intestines tissue of the wild (W) and farmed (F) lenok (*Brachymystax lenok*). Results are shown as Mean ± SD (*n* = 5). Values marked with asterisks are significantly different (**P < 0.01).

The qRT-PCR results demonstrated significant transcriptional differences in immune- and inflammation-related genes between wild and farmed *B. lenok* (Figures 6, 7). In liver tissue, the wild group showed markedly elevated mRNA expression levels of *c3, foxo1, igM, il-6, il-10, il-1β, tlr1, tlr3, tnf-α, lyz, mpo, myd88*, and *ssa1* compared with the farmed group (*P* < 0.05). In intestinal tissue, mRNA expression levels of *c3, foxo1, igM, il-10, lyz, mpo, nf-κb, ssa1, tlr1, tlr3*, and *tnf*-α were significantly upregulated in wild *B. lenok*, whereas *il-6, il-1*β, and *myd88* were significantly downregulated (*P* < 0.05).

### 3.7 Comparative analysis of intestinal microbial composition in wild and farmed populations

**FIGURE 8:**
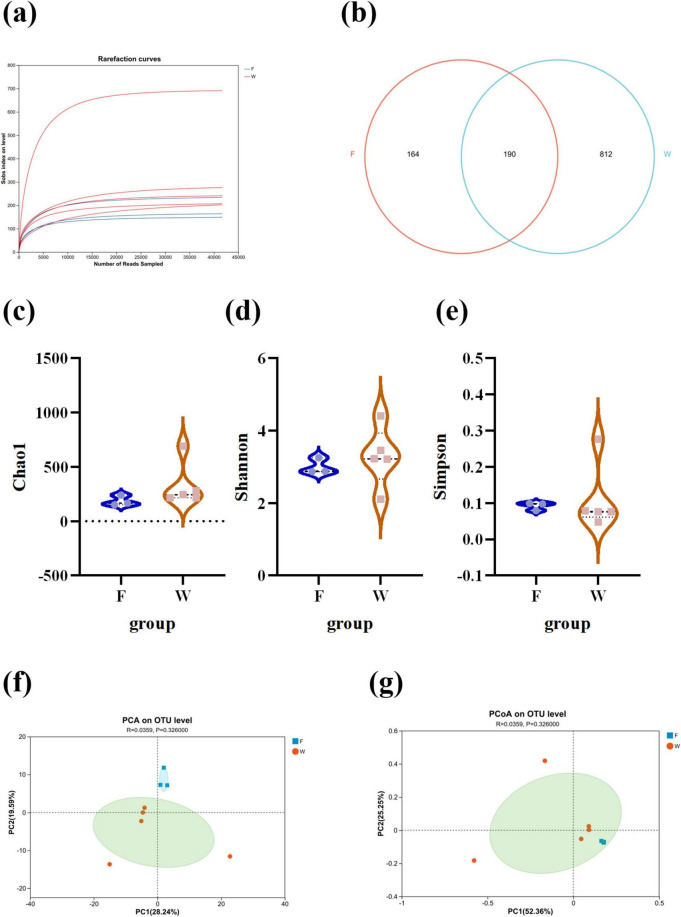
The intestinal microbe changes in lenok (*Brachymystax lenok*) under wild and farmed environments. **(a)** Rarefaction curve. **(b)** Venn diagram of operating taxonomic units. **(c–e)** α-diversity analysis for the intestine microbiota of the wild (W) and cultured (F) *Brachymystax lenok*. **(f–g)** Comparisons of β-diversity of the gut microbiota between the wild (W) and captive (F) *Brachymystax lenok*. F, farmed group; W, wild group.

**FIGURE 9:**
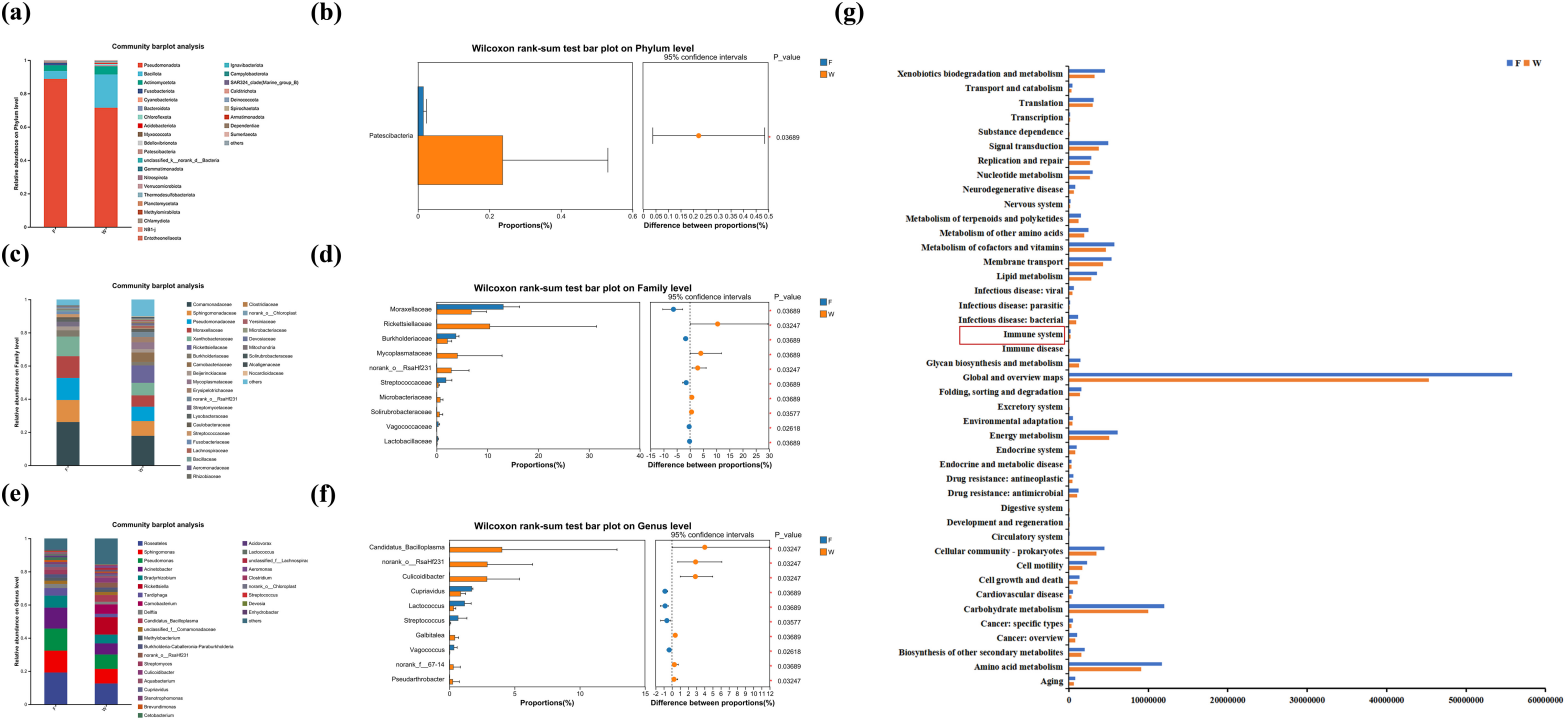
Differences in the relative abundance of the intestine bacteria flora of wild (W) and farmed (F) lenok (*Brachymystax lenok*). **(a)** Relative abundance of intestinal bacteria at the phylum level. **(b)** The significant bacterial community abundance at the phylum level. **(c)** Relative abundance of intestinal bacteria at the family level. **(d)** The significant bacterial community abundance at the family level. **(e)** Relative abundance of intestinal bacteria at the genus level. **(f)** The significant bacterial community abundance at the genus level. **(g)** KEGG pathways are ample or depleted in the intestinal microbiota of wild (W) and farmed (F) lenok (*Brachymystax lenok*).

Rarefaction curve analysis (Figure 8a) showed that the Sobs index plateaued with increasing sequencing depth, indicating that the sequencing coverage was sufficient to reflect microbial diversity in all samples. A total of 1,166 operational taxonomic units (OTUs) were identified across all samples. Among them, 812 OTUs (69.64%) were unique to the wild group, 164 OTUs (14.07%) were unique to the farmed group, and 190 OTUs (16.30%) were shared between both (Figure 8b). Alpha diversity analysis (Figures 8c–e) revealed similar richness and evenness between the wild and farmed groups, with no significant differences in the Chao1, Shannon, or Simpson indices (*P* > 0.05). Beta diversity analysis based on Bray-Curtis algorithm, along with PCA and PCoA visualization, confirmed no significant differences between groups (*P* > 0.05) (Figures 8f,g). At the phylum level, the gut microbiota of both wild and farmed *B. lenok* were dominated by Proteobacteria and Firmicutes (Figure 9a). In wild *B. lenok*, Firmicutes, Actinomycetota and Bacteroidetes increased by 15.23, 1.52, and 0.20%, respectively, while Proteobacteria, Fusobacteriota, and Cyanobacteriota increased by 17.3, 1.57, and 0.33%, respectively, compared with farmed fish. Additionally, Patescibacteria abundance was significantly higher in wild *B. lenok* (*P* = 0.04) (Figure 9b). At the family level, the dominant taxa in wild *B. lenok* included Comamonadaceae (17.83%), Sphingomonadaceae (8.88%), Pseudomonadaceae (8.67%), and Moraxellaceae (6.83%), while the farmed group was dominated by Comamonadaceae (26.15%) and Pseudomonadaceae (13.31%) (Figure 9c). Moraxellaceae and Burkholderiaceae were significantly more abundant in farmed *B. lenok* (*P* = 0.04), whereas Rickettsiellaceae and Mycoplasmataceae were significantly enriched in wild individuals (*P* = 0.03; *P* = 0.04, respectively) (Figure 9d). At the genus level, both groups were dominated by *Rhodobacter* (Figure 9e). However, *Candidatus_Bacilloplasma*, *norank_o_RsaHf231*, and *Culicoidibacter* were significantly more abundant in wild *B. lenok* (*P* = 0.03), whereas *Cupriavidus*, *Lactococcus*, and *Streptococcus* were significantly more abundant in the farmed group (*P* = 0.04) (Figure 9f). *In silico* functional prediction via PICRUSt2 suggested potential alterations in microbial metabolic pathways related to energy metabolism, carbohydrate metabolism, lipid metabolism, amino acid metabolism, and immune system functions between groups. These predictions represent putative functional capacities derived from 16S rRNA gene sequences and require experimental validation (e.g., metagenomic or metabolomic analyses) to confirm actual pathway activity (Figure 9g).

## 4 Discussion

In this study, we investigated the differences in intestinal microbial composition, digestive enzyme activity, intestinal structure, and immunity between wild and farmed *B. lenok*, and simultaneously explored the contribution of the digestive–immune–microbiome synergistic adaptation mechanism to the establishment of adaptability under different environments. *B. lenok* is currently endangered due to factors such as anthropogenic disturbances and habitat degradation. Artificially hatching fish and reintroducing them into the wild is an effective method to supplement wild germplasm resources. However, efforts to enhance stocks of *B. lenok* have achieved poor results. Studies have shown that the loss of wild adaptive traits in farmed fish is an important reason why stocked fish fail to survive in a wild environment (Willoughby et al., 2015). Composition of the intestinal microbiota is an adaptive trait and is particularly important for *B. lenok*. Our study found that environmental conditions significantly influenced gut microbial community structure. Similar to our findings in *B. lenok*, recent studies on duck gut microbiome have shown that rearing conditions significantly influence the composition and function of gut microbiota (Ma et al., 2024). This highlights the importance of rearing conditions in shaping the gut microbiome and its potential impact on host health. This likely reflects the broader range of natural food sources available in the wild, with natural environments providing diverse nutrients that require a more complex microbial community for digestion (Diwan et al., 2023; Chi et al., 2019).

A growing body of evidence indicates that the nature of the diet shapes the composition of the gut microbiota in different hosts (Ross et al., 2024). In this study, we further revealed variations in gut microbial composition between farmed and wild *B. lenok*, with both groups characterized by dominance of Proteobacteria and Firmicutes. However, unlike in the farmed group, Firmicutes showed an increasing trend while Proteobacteria exhibited a decreasing trend in the wild group. Studies have demonstrated that members of Firmicutes play important roles in the metabolism, digestion, and absorption of proteins and other nutrients (Chen et al., 2022). Firmicutes are involved in fatty acid breakdown, with their primary functions including providing nutrients and energy to epithelial and gastrointestinal cells, promoting mucus production, and exerting anti-cancer and anti-inflammatory effects. Their depletion may impair intestinal barrier integrity (Hao et al., 2017; Zhou et al., 2023). Similarly, Bacteroidetes can also enhance digestion, decompose polysaccharides and proteins, improve the utilization efficiency of assimilated nutrients, and maintain intestinal microecological balance (Zhang et al., 2025). In this study, the increased abundance of Firmicutes and Bacteroidetes in wild *B. lenok*, on the one hand, indicates that the gut microbiota of *B. lenok* in a wild environment can help the host utilize nutrients more efficiently and harvest energy from food. In the wild, due to food scarcity and reduced intake of proteins and fats, the increased content of Firmicutes enhances nutrition and promotes intestinal health in *B. lenok*. On the other hand, as observed in many animals, diet is a key factor influencing the gut microbiota (Hills et al., 2019). Therefore, the gut microbiota of farmed *B. lenok* exhibits a higher level of carbohydrate metabolism, as high-carbohydrate and high-fat feeds are widely used in aquaculture practices (Xie et al., 2017). The study also found that “carbohydrate metabolism” and “lipid metabolism” were both increased in the KEGG pathways of the farmed group. Wild *B. lenok* primarily feed on small protein-rich fish and benthic organisms, thus requiring a high level of Bacteroidetes to utilize proteins for energy supply in normal metabolic processes. We hypothesize that this diet reflects a close match between the species’ nutritional profile and the available local prey resources, thereby minimizing foraging costs. Moreover, the abundance of benthic organisms aligns well with the nutritional niche of *B. lenok* in its natural habitat. Considering that the different dietary compositions between farmed and wild *B. lenok* significantly affect the composition of the gut microbiota. Thus, we suggest that artificially hatched *B. lenok* should be provided with a diet and living environment similar to those of wild populations before being reintroduced into the wild. This will help *B. lenok* adapt more quickly to the wild environment and diet after release.

The intestine is a key site for digestion and absorption in fish, and the integrity of intestinal structure is crucial for fish digestion, absorption, growth, and development (Zhu et al., 2025). The intestines of both wild and farmed *B. lenok* consist of four layers (mucosa, submucosa, muscularis, and serosa) and contain intestinal glands. Studies have shown that fish intestinal villi are located on the surface of the intestinal mucosa, formed by the protrusion of mucosal epithelium and lamina propria into the intestinal lumen. Their function is to increase the inner surface area of the intestine, thereby improving food digestion and absorption rates (Cheng et al., 2012; Meirelles et al., 2021). When the length and width of intestinal villi increase, the absorption capacity of the intestine also enhances (Wang J. et al., 2025). Based on these morphological divergences, we propose the following working hypotheses regarding functional adaptations: In this study, shorter villi in foregut of wild *B. lenok* may reduce the absorptive surface for the low-starch, high-protein/high-fat natural prey, potentially minimizing post-prandial ROS exposure. Longer villi and enlarged submucosa in hindgut of wild fish could expand the surface for final amino-acid uptake and SCFA absorption from trace fermentable chitin. Conversely, farmed fish receiving high-fat pellets (20% lipid) show elongated villi in foregut that may maximize glucose uptake, but shorter villi in hindgut and thinner walls, suggesting adaptation to continuous pellet flow and reduced mechanical demand. This indicates that the anterior intestine enhances the digestion and absorption of natural bait in wild *B. lenok*. The thickness of the muscularis is an important indicator reflecting intestinal contractile capacity. An increase in muscularis thickness helps improve the capacity of the intestine to digest its contents, thereby enhancing growth performance (Li et al., 2014). In this study, the muscularis thickness of the anterior, middle, and posterior intestines of wild *B. lenok* was significantly increased compared with that of the farmed group, which may be related to the physical properties of their diet. Wild *B. lenok* is a carnivorous fish that primarily feeds on benthic organisms. Their prey have hard exoskeletons and bones, so a thicker muscularis is required to enhance intestinal peristalsis and promote food grinding and emptying.

Digestive enzymes are a type of enzyme in fish; they are special proteins that can decompose macromolecular organic substances into small molecular substances for absorption by the body, and they play important roles in fish feeding, digestion, absorption, and metabolism (Amenyogbe et al., 2024; Krogdahl et al., 2005). Compared with farmed *B. lenok*, the intestinal trypsin activity of wild fish was significantly reduced, while the gastropancreatic trypsin level remained stable. The peaks of trypsin in both groups were located in the pyloric caeca and mid-intestine, but the lower intestinal protease activity in wild *B. lenok* indicates that natural prey (fish and benthic organisms) provide highly digestible proteins, thereby reducing the demand for pancreatic secretion of digestive enzymes. In contrast, farmed fish fed pellet feed require upregulation of intestinal trypsin to compensate for the insufficient digestibility of raw materials due to the high content of plant proteins and anti-nutritional factors (Sarwar Gilani et al., 2012). Amylase activity was significantly higher in the livers and stomachs of wild *B. lenok* compared with the farmed group, but lower in the mid-intestine. One possible explanation for the elevated posterior-intestine amylase activity in farmed *B. lenok* involves the enrichment of lactic acid bacteria (LAB) *Lactococcus* and *Streptococcus*, which were identified as signature taxa in the farmed group. These genera have been reported to secrete extracellular amylases and produce lactate through carbohydrate fermentation (van Asseldonk et al., 1993; Ghailan and Niamah, 2025; Ray et al., 2012), potentially aiding in the degradation of complex carbohydrates (e.g., starch) in commercial feeds. Additionally, microbial lactate production might lower local pH, which could further activate host digestive enzymes (Petrov et al., 2008; Liu et al., 2020). However, we acknowledge that this interpretation remains speculative; elevated amylase activity could equally reflect diet-induced host plasticity (direct upregulation by high-starch pellets) rather than microbial stimulation. Direct experimental evidence (e.g., germ-free vs. colonized fish) is required to establish causality. Studies have shown that the surge in amylase activity in liver and stomach tissues is consistent with a carnivorous strategy: natural prey has extremely low starch content, and fish glycogen is hydrolyzed first at the pylorus, avoiding energy consumption in the mid-intestine; the peaks in the posterior intestines of both wild and farmed *B. lenok* reflect the final hydrolysis of residual disaccharides, while the higher liver amylase in the wild group may also support glycogenolysis during burst swimming when hunting prey (Wu, 2017; Goertzen et al., 2012). In our study, lipase showed the clearest habitat characteristics. The activity of lipase in the livers and pyloric caeca of wild fish was significantly higher than that in the farmed group, while the activity in the posterior intestine was lower. This indicates that the anterior part of the digestive system in wild *B. lenok* efficiently decomposes lipids to obtain energy from high-fat prey; the decreased enzyme activity in the posterior part of the digestive system suggests that lipolysis can be completed in the anterior part to reduce energy loss. Farmed fish maintain high lipase levels in the posterior part of their digestive systems to recover residual fat due to high-fat pellet feed (Yuan et al., 2025). Moreover, this explains the lower lipase activity in the posterior part of the digestive system in wild fish. In conclusion, the increased amylase and lipase in the anterior part of the digestive system, decreased intestinal trypsin, and low enzyme activity in the posterior part of the digestive system in wild *B. lenok* constitute a unique enzyme distribution pattern that matches that of their natural high-protein, high-fat, and low-starch prey, thereby improving growth efficiency in fluctuating wild environments.

As an important digestive organ, the intestine not only absorbs nutrients from food, but also participates in various immune responses in the body (Egerton et al., 2018; Wang et al., 2024). Among them, the lamina propria is the primary site of the intestinal immune response, containing many immune cells. It plays important roles in regulating intestinal homeostasis, inducing immune tolerance, and resisting pathogenic bacterial infections (Zhang et al., 2024). In this study, the thickness of the lamina propria in the anterior and mid-intestines of wild *B. lenok* was significantly increased (*P* < 0.05). Patescibacteria (significantly more abundant in wild *B. lenok*) is typically associated with nutrient-limited environments and is hypothesized to rely on host or other microbial taxa for essential nutrients (Tian et al., 2020). In fish, Patescibacteria may play a role in modulating the gut microenvironment by competing with potential pathogens for resources, thereby indirectly enhancing host immunity (Srinivas et al., 2024). This means that wild *B. lenok* have stronger immunity, which may be necessary for survival in complex wild environments. In addition, studies have confirmed that an increase in the abundance of Proteobacteria may be a potential diagnostic marker for metabolic disorders and immune dysregulation (Litvak et al., 2017). Proteobacteria are also considered the dominant phylum in many farmed fish, such as rainbow trout (*Oncorhynchus mykiss*) and Atlantic salmon (*Salmo salar*) (Li et al., 2023; Gajardo et al., 2016). A study by Zhao et al. (2019) showed that the abundance of Pseudomonas significantly increased after immunosuppression. In this study, the expression levels of immune-related genes, such as *il-10*, *c3*, *foxo1*, and *igM*, in the livers and intestines of farmed *B. lenok* were significantly lower than those in the livers and intestines of the wild group. Therefore, we speculate that Proteobacteria may reduce the expression of immune-related genes in the liver and intestine of farmed *B. lenok* by suppressing immune responses. While this correlation might suggest potential immunomodulatory effects of these bacteria, we acknowledge that this pattern could alternatively reflect confounding aquaculture conditions, including (i) dietary differences (diet altering both microbiota and immune status), (ii) chronic stress (handling/confinement immunosuppression), (iii) high rearing density (crowding stress and pathogen load), or (iv) distinct water microbiome exposure, rather than direct bacterial inhibition of host immunity. Direct experimental evidence is required to distinguish these possibilities.

In addition to immune-related genes, we also determined the activity of related immune enzymes. AKP, ACP, and LZM are key indicators of non-specific immunity in fish (Li et al., 2021; Zheng et al., 2022), and differences in their tissue distribution can reflect the allocation of immune resources under different life history strategies. In this study, the activities of AKP, ACP, and LZM in the livers of wild *B. lenok* were significantly higher than those in the livers of farmed individuals, while the activities of the three enzymes in the intestine showed the opposite trend. This “liver-biased” immune enhancement indicates that wild fish upregulate non-specific defense enzymes (AKP/ACP/LZM) in the liver to combat more diverse environmental antigens and potential pathogens in the water. Moreover, the relative decrease in intestinal enzyme activity reflects the lower antigenic pressure generated by *B. lenok*’s high-digestibility natural prey (fish, benthic organisms). In contrast, farmed fish rely on high-fat and high-plant protein pellet feed, and the intestine must maintain higher levels of AKP, ACP, and LZM to eliminate anti-nutritional-factor-related antigens and prevent excessive bacterial proliferation. Similar results have been reported by Wang X. et al. (2025). Our results indicate that tissue-specific immune allocation is an important physiological mechanism for enabling *B. lenok* to adapt to different habitats.

Interestingly, a similar pattern was observed in the results for the detection of antioxidant indicators. The antioxidant enzymes CAT and GSH in the liver showed a trend of being significantly higher in wild than farmed *B. lenok*, but in the intestine, CAT activity and MDA content were higher in farmed than wild *B. lenok*. During normal metabolic processes, the dynamic balance of the redox system keeps the body in a stable state. When fish are subjected to internal and external environmental stresses, reactive oxygen species (ROS) are produced in the body, leading to an imbalance in the antioxidant system, which ultimately results in decreased intestinal health, weakened immunity, and reduced growth performance (Hoseinifar et al., 2020; Kakade et al., 2020). CAT, GSH, and MDA are important indicators for evaluating antioxidant function. Among them, CAT and GSH can scavenge free radicals, reduce oxidative stress-induced damage to the intestinal mucosa, and play key roles in intestinal defense and repair (Tunç et al., 2025; Joy et al., 2017). Meanwhile, MDA content reflects the degree of lipid peroxidation, indirectly indicating the degree of cell damage (Zhou et al., 2025). These results suggest that wild individuals upregulate their liver antioxidant systems (CAT/GSH) to scavenge the higher ROS load in fluctuating natural water, while the increase in CAT and accumulation of MDA in the intestines of farmed fish reflect local oxidative stress induced by high-fat pellet feed, which requires continuous mobilization of antioxidant defense to maintain intestinal barrier integrity (Chen et al., 2023). The higher intestinal MDA in farmed fish aligns with the 20% fat content of the commercial diet. Moreover, it indicates that farmed and wild *B. lenok* deploy antioxidant enzymes differentially to match the oxidative pressure of different habitats, thereby optimizing energy utilization and enhancing environmental adaptability.

## 5 Conclusion

In summary, this study revealed significant differences between wild and farmed *B. lenok* in terms of intestinal microbiota composition, digestive enzyme activity, immune responses, and antioxidant capacity. The differential distribution of digestive, antioxidant, and immune enzymes across liver and intestinal tissues represents key physiological adaptations that enable *B. lenok* to cope with oxidative stress, optimize energy utilization, and enhance environmental adaptability. We propose that pre-release acclimation strategies, such as feeding hatchery fish natural high-protein diets, may help restore liver-centered immunity and foregut-dominant digestive enzyme patterns. To test this hypothesis, we propose a controlled pilot study (*n* ≥ 15 per group): (i) Acclimation Phase: Hatchery-reared *B. lenok* (current pellet diet) randomly assigned to (a) gradual dietary transition (25%/50%/75%/100% natural prey) over 4–6 weeks, or (b) continued pellet control; (ii) Monitoring Endpoints: Serial sampling (pre-transition, mid-transition, pre-release) to assess (a) gut microbiome succession (16S rRNA), (b) hepatic immune gene expression (qPCR), (c) digestive enzyme zonation (amylase/lipase activity in foregut vs. hindgut), and (d) histological gut remodeling (et., villus length); (iii) Release Outcomes: Post-release survival and microbiome convergence to wild-type profiles at 1/3/6 months as ultimate success metrics. Only if pilot data demonstrate significant microbiome/physiological convergence to wild phenotypes should larger-scale pre-release conditioning be considered. These measures could improve the survival rates of hatchery-reared *B. lenok* after reintroduction. The findings of this study provide a theoretical foundation for healthy aquaculture management and conservation-oriented reintroduction programs for *B. lenok*. This study suggests that aquaculture practices may restructure the gut microbiome and host physiology of *B. lenok*, though larger cohorts are needed to establish the generality of these patterns.

## Data Availability

The datasets presented in this study can be found in online repositories. The names of the repository/repositories and accession number(s) can be found below: https://www.ncbi.nlm.nih.gov/bioproject/PRJNA1425741.
