# Supplementary material for: Comprehensive evaluation of environment adaptability in wild and captive lenok (Brachymystax lenok): from the perspective of antioxidant capacity, immune response and gut microbiome
Source: Front Microbiol. 2026 Mar 4;17:1764670. doi: 10.3389/fmicb.2026.1764670 (PMC12996260; doi:10.3389/fmicb.2026.1764670)
Supplement: Supplementary file 1 [file Table_1.docx]

**Table S1. The nutrient content of feed for *Brachymystax lenok***

| Nutrient content indicators (g/100g) | |
| --- | --- |
| Crude protein | 43.4 |
| Crude fat | 20 |
| Crude fiber | 3 |
| Crude ash | 9 |
| Water content | 8.5 |
| Total Phosphorus | 12 |
| Ca | 0.8 |
| Amino acid | 3.3 |

**Table S2. Water quality parameters of wild and farmed environments**

|  | Wild group | Farmed group |
| --- | --- | --- |
| Water temperature | 9.1 ± 0.64℃ | 9℃ |
| Dissolved oxygen (DO) | 7.7 ± 1.2 mg/L | 7.2 ± 0.3 mg/L |
| pH value | 6.5 ± 1.06 | 6.8 ± 0.12 |
| Ammonia nitrogen | 0.05 mg/L | 0.025 mg/L |

**Table S3. Complete weight data for each prey category**

| **Prey items** | **Weight**% |
| --- | --- |
| Benthic animals | **44.88** |
| Ephemerella sp. | 0.01 |
| Heptagenia sp. | 1.83 |
| Oyamia sp. | 0.92 |
| Pteronarcys sachalina | 17.32 |
| Pteronarcys sp. | 7.38 |
| Rhyacophila sp. | 0.98 |
| Hydropsyche sp. | 0.05 |
| Stenopsyche sp. | 4.95 |
| Ceraclea tsudai | 1.99 |
| Psilotreta sp. | 3.96 |
| Limnophilus sp. | 3.99 |
| Nephrotoma sp. | 0.01 |
| Hybomitra hirticeps | 0.02 |
| Philorus sp. | 0.01 |
| Rhantus suturalis | 0.02 |
| Stylogomphus shirozui shirozui | 0.43 |
| Davidius sp. | 0.25 |
| Diplonychus sp. | 0.76 |
| Terrestrial insect | **16.49** |
| Pteronarcus sp. | 16.19 |
| Cerambycidae sp. | 0.3 |
| Fish | **38.6** |
| Phoxinus phoxinus | 0.88 |
| Phoxinus lagowskii | 37.78 |
